# Strategies and governance to reduce health inequalities: evidences from a cross-European survey

**DOI:** 10.1186/s41256-017-0038-7

**Published:** 2017-07-03

**Authors:** Sara Barsanti, Louis-Rachid Salmi, Yann Bourgueil, Antonio Daponte, Ewelina Pinzal, Solange Ménival

**Affiliations:** 1 Laboratorio Management e Sanità, Institute of Management, Scuola Superiore Sant’Anna, Pisa, Italy; 2 Institut de Santé Publique, Épidémiologie et de Développement, INSERM U-1219 Bordeaux Population Health Research Center, Université de Bordeaux et CHU de Bordeaux, Pôle de Santé publique, Bordeaux, France; 3 Institut de Recherche et de Documentation en Economie de la Santé, Paris, France; 4 Observatorio de Salud y Medio Ambiente de Andalucía (OSMAN), Escuela Andaluza de Salud Pública, Granada, Spain; 5 Conseil Régional d’Aquitaine, Bordeaux, France

**Keywords:** Health Inequality, Governance System, Action Spectrum, Health Equity, Variable Situation

## Abstract

**Background:**

The main objective of the paper is to identify the governance system related to policies to reduce health inequalities in the European regions. Considering the Action Spectrum of inequalities and the check list of health equity governance, we developed a survey in the framework of the AIR Project - Addressing Inequalities Intervention in Regions - was an European project funded by the Executive Agency of Health and Consumers.

**Methods:**

A web-based qualitative questionnaire was developed that collected information about practiced strategies to reduce health inequalities. In total 28 questionnaires from 28 different regions, related to 13countries, were suitable for the analysis.

**Results:**

Progress in health equity strategies at the national and regional levels has been made by countries such as France, Portugal, Poland, and Germany. On the other hand, Spain, Italy, and Belgium have a variable situation depending on the region. However, the results of the survey indicate that the governance system for health equity different in terms of commitment, resources and tools.

**Conclusions:**

The survey highlights a weakness of governance system for the majority of countries in terms of evaluation actions and of impact of interventions in reducing inequalities, and the difficulties in having a clear and integrated vision between the national and regional levels.

**Electronic supplementary material:**

The online version of this article (doi:10.1186/s41256-017-0038-7) contains supplementary material, which is available to authorized users.

## Background

Pursuing equity in health can be defined as the act to eliminate differences in health between population groups, such as between rich and poor, that are considered unfair, unjust and avoidable [1]. In this sense, health inequalities are defined as systematic differences in health that can be avoided by appropriate policy intervention and that are therefore deemed to be unfair and unjust [2]. There are three critical assets to evaluate an equitable health system [3]: i. Equal access to health care for those in equal need of health care; ii. Equal utilization of health care for those in equal need of health care; iii. Equal (or, rather, equitable) health outcomes (as measured by, for example, quality adjusted life expectancy). The first and second points are related to the concept of equity of health care, in terms of access, utilization and quality; the third point refers to the concept of equity in health.

Equity simultaneously requires that relevantly similar cases must be treated in similar ways, and relevantly different cases in different ways. Aday et al. [4] define an “equitable distribution of healthcare services” as “one in which illness (as defined by the patient and his family or by health-care professionals) is the major determinant of the allocation of resources.” In terms of health service provision, this requires a well-balanced combination of strong universal mainstream health services that are accessible and responsive to the special needs of specific population groups, as well as targeted health services for particular groups to meet major health issues [5]. For example, as Mackenbach underlines [6, 7], in almost all European countries, the rates of death and poorer self-assessments of health were substantially higher in groups of lower socioeconomic status. Furthermore, the magnitude of the inequalities between groups of higher and lower socioeconomic status was much larger in some countries than in others (i.e. inequalities in mortality were small in some southern European countries and very large in most countries in the eastern and Baltic regions, while socio-economic inequalities in obesity are more pronounced in Southern Europe than in the Eastern and Baltic regions of Europe). Considering the European Commission [8], educational gradients in life expectancy existed in all Member States and they vary by sex, age and the overall level of survival. Life expectancy at age 25 for men with tertiary education in Estonia was 17.8 years longer, or 50% higher, than life expectancy for men who did not complete secondary education; for Hungary were 13.3 years and 34%. In Malta, Norway, Sweden and Italy the differences between the same two groups ranged from 3.2 to 5.2 years, which is 6–10%.

Different theoretical models have been proposed to explain the effect of social factors on health. Most are based on the idea that social position affects health status. Similarly, another model links social position to health risk factors exposure in the environment or in lifestyle. A frequently used framework is the one proposed by Dalgren & Whitehead in 1991 [5]. The framework describes health determinants in 5 layers, including social factors and relations between the different determinants. In this framework, material circumstances including access to health care can be used as action target. The contribution of social determinants to health inequalities is a complex issue and it also differs greatly from country to country and region to region. Most senior public health policy-makers agree that specific interventions are needed to reduce social inequalities in health. However, which interventions are most effective in reducing the observed inequalities are not well understood [9]. However, some authors [10], suggest the robust evidence that some public health intervention types increase inequalities between socioeconomic groups (i.e. media campaigns). Interventions such as resource provision, fiscal interventions and structural workplace have several intervention types appear promising in reducing inequalities between socioeconomic groups. This has important implications for those seeking to develop, implement and evaluate public health interventions, whether they explicitly aim to reduce inequalities or not.

The development of policies to tackle health inequalities should therefore be guided by country- and region-specific analyses that determine what interventions offer the best potential to narrow the country- or region-specific health gaps between particular socio-economic groups [6, 7]. However, many researchers highlights that good intentions of national policy to reduce health inequalities have often not translated into improved health outcomes for all [11–13]. In order to understand why priorities remain often at the level of good intentions and are not always clearly translated into specific projects, many authors [14, 15] consider necessary to explore how well governing for equity in health through action on social determinants is being carried out. The aim of this paper is describing the governance for health equity adopted by European countries considering the results of an European project -The Addressing Inequalities in Regions (AIR) - a European project funded by the Executive Agency of Health and Consumers [16, 17]. In particular, the paper discusses the health care system equity governance considering the main framework related to the governance for health equity check list describe by Margaret Whitehead [18] and Brown et al. [14] in order to monitor each country on a set of variable. On the basis of the country profile towards the checklist related to the governance for health equity, we update the position of each countries in the “*action spectrum of inequalities*”, developed by Whitehead in 1991 [19], by which European countries were categorized at different levels of awareness and concern toward health inequalities through the “action spectrum of inequalities”.

At the core of this concept is recognition that complex issues – such as problems that have no simple solution or to which the solution cannot be found through research alone (for example, issues surrounding inequities) – require new system-based governance approaches [20, 21]. Such approaches are capable of addressing the interdependencies of factors (determinants, stakeholders, settings) that are part of the causal chain and necessary for achieving sustainable solutions [14].

Next part will discuss the framework and the position of a set of European countries in previous studies; than the following part will analyze AIR results based on the governance for equity conceptual theory, with a brief summary on AIR methodology and survey. The last part will provide a discussion on health inequalities strategy in the European countries, considering possible developments and improvements in terms of governance and interventions.

### The governance for health equity and the action spectrum of inequalities

Although policies directly targeting health inequalities have focused in the past decade on the poorest part of the population living in the most precarious conditions (the gradient dimension of the health inequalities issue is not considered globally as a target of policies), and have been directed mainly at facilitating access to health care, the situation on health inequalities strategies and interventions still differs from country to country. The context and content of such policies vary markedly across these systems, reflecting different political ideologies and historical, social, and political legacies in each country [22].

Margaret Whitehead [19] suggests that countries can move along the “*Action Spectrum of Inequalities* (ASI)”, which refers to different steps in considering the political attitude towards health inequalities, from measuring health inequalities to recognition of disparities and an awareness of health determinants and the consequences.

Once awareness is raised, there may be concern, denial, or indifference about inequalities. If there is concern, the countries can develop a will to take action and move through a process from isolated initiatives to more structured developments, and ultimately to a comprehensive and coordinated policy. In this sense, diffusion refers to the process by which research evidence and awareness of seriousness of the issue of social inequalities in health have come to attention to European policy makers at both national and international levels,

According to Whitehead, individual countries have raised awareness on the issue of health inequalities through very different methods, according to the dictates of prevailing circumstances and political climate. Staring from the 1990s, different researches used the ASI to monitor each European country with respect to health inequalities strategies diffusion. Following this framework, the report ‘*Health inequalities: a Challenge for Europe*’, commissioned by the UK Presidency of the EU in the 2006 [23], divided the European countries into four main groups:

- Group A: countries where there was no type of action on the reduction of inequalities, such as Cyprus and Greece.
- Group B: countries with isolated initiatives on the reduction of inequalities but with no national strategy, such as Belgium, France, Germany, and Poland.
- Group C: countries with a clear strategy for reducing inequalities within a broader policy to promote health, such as The Netherlands, Finland, Denmark, and Hungary.
- Group D: countries with an integrated plan to reduce inequalities in health, such as England, Scotland, Ireland, and Sweden.

The EU in evaluating the policies related to health inequalities identified three country clusters, with a focus on the existence (or lack thereof) of national-level HI-focus policies, and whether countries’ policies explicitly or implicitly responded to health inequalities [8]: Cluster 1 — Relatively positive and active response to health inequalities; Cluster 2 — Variable response to health inequalities; Cluster 3 — Relatively undeveloped response to health inequalities. However, this analysis do not consider some related issue on terms of governance system. Furthermore, to better understand different approaches of countries and where the strategies have been comprehensive and coordinate, it is necessary to look not only at policy responses, but also at the ways those policy decisions are being made, implemented and reviewed: that is, to explore how well governance system for equity in health is being carried out. The term governance refers to “*the institutions, rules and norms through which policies are developed and implemented – and through which accountability is enforced. (…) However, governance is not just about abstract institutional processes or formal rules. It is also about power relationships in society*” [24]. A governance system in health care [20, 21] is necessary to build a comprehensive health equity approach, to ensure joint action and accountability of health and non-health sectors and to strengthen health care and health equity policies in order to improve outcomes for all the population. Moreover, governance for health equity has an important role to play in order to: “*i. develop the necessary legislation and regulations to strengthen joint accountability for equity, across sectors and decision-makers and within and outside of government; ii. use mechanisms which actively promote involvement of local people and stakeholders in problem definition and solution development; iii. ensure regular joint review of progress, which fosters common understanding and sustains commitment to deliver shared results over time; iv. draw on different forms of evidence to ensure policies address the main causal pathways and are capable of adapting over time*” [19]. Brown et al. [14] set out a systems check list for health equity governance, described in Table 1. The governance system proposed considers eight different domains and functions which need to be embedded in the governance arrangement of a country in order to deliver improved equity in health.

**Table 1:** Governance domains and system characteristics

Domain	Systems characteristics
1. Political commitment	Clear political commitment in terms of national and regional plan and strategies
2. Intelligence	Evidence and information on health inequities and SDH to: • inform policy and investment decisions • monitor progress • hold stakeholders to account
3. Accountability structures and systems	Legislative structures and systems enabling intersectoral action on equity and SDH at European, national and local levels. Statutory governance boards capable of holding all stakeholders to account. Legislative structures and systems: (i) enabling formation and action of NGOs and civil society groups as partners in action to reduce inequities; and (ii) monitoring progress
4. Policy coherence across government sectors and levels	Formal framework setting out stakeholders involved in action for improving equity in health Framework linked to ministerial portfolios and budgets, nationally and locally. Government policy audited through health impact assessment and equity impact assessment. Instruments that institutionalize collaboration across sectors and levels of government.
5. Involving local people	Commitment to participation of local people and subnational authorities in policy design and review. Instruments and systems that secure community involvement in solutions. Intelligence and data on health, equity and SDH made accessible within the public domain – locally, nationally and across Europe.
6. Institutional and human resource capacity	Capacity development, including: • development of competent and trained staff • institutional processes
7. Modernized public health	Review of public health training and practice
8. Learning and innovation systems	Commitment to continuous improvement in understanding the efficacy of policies and interventions to reduce inequities. Commitment to ongoing performance review/improvements in governing for equity in health, through action on SDH

In the contest of health equity, a good governance system could imply a relative good position of the institution (national, regional or level) in the action spectrum of inequality [19]. The check list is based on the key attributes against which ‘good’ governance is appraised, that are [25, 26]: 1. Legitimacy and Voice, in terms of participation and collaboration; 2. Direction, in terms of a clear and long-term strategic vision; 3. Performance, in terms of responsiveness, effectiveness and efficiency; 4. Accountability, considering measurement and free flow-information; 5. Fairness that include both the equity rationale and legal frameworks. Different levels and approaches with regards the governance system for health equity may reflect different involvement of government and resources and consequently different position of the countries in the ASI. The aim of the paper is to analyze the governance system at both at European level and national level, considering the results of a European survey on strategies and interventions for reducing health inequalities [16, 17]. Table 1 summarize the eight domains and the check list proposed by Brown [14] and related issues and proxies in order to measure the governance system related to equity in Europe. For each of the domains proposed by Brown [14], we specified one or more questions that act as a proxy measure to express the governance equity related system, as shown in Table 2.

**Table 2:** Regions and Countries

Region	Country
1. Wien; 2. Vorarlberg	Austria
3. Welzijn, Volksgezondheid en Gezin;4. Wallonia; 5. Vlaams Gewest	Belgium
6. Aquitaine; 7. Region Centre France; 8. Limousine	France
9. Magdebourg; 10. Sassonia	Germany
11. Umbria; 12. Trento; 13. Piemonte; 14. Toscana	Italy
15. Lubuskie; 16. Slask; 17. Malopolskie; 18. Swietokrzyskie	Poland
19. Algarve; 20. Lisboa et Vale do Tejo; 21. Azzorre; 22. Madeira	Portugal
23. Valencia; 24. Sevilla	Spain
25. Limburg-Noord; 26. Zuid-Holland Zuid	The Netherlands
27. East Midlands; 28. East of England	United Kingdom

## Methods

The AIR Project has been described elsewhere [16, 17]. All partners of the AIR project are listed in the Additional file 1. This article is focused on the analysis of a qualitative web-based survey on health equity strategies at regional level. The need for such a survey resulted from the first part of the AIR Project, a bibliographic research, which was considered not exhaustive, as there are many interventions that only health operators, regional and local, are aware of, that cannot be described in the literature. The questionnaire collected information about strategies, actors and tools that are involved in the reduction of health inequalities in EU regions or through policies implemented at the national, regional, or local levels. In this perspective, the questionnaire represented an opportunity to gather qualitative information on governance system for ensuring equity in health in regions and countries.

The developed questionnaire had about 30 structured questions in total, of which 95% were closed questions and 5% open.

The questions taken into consideration for the analysis concern the main points that reflect the governance system for health equity implemented in a country. This framework “Governance domains and relative questions” considers 8 different dimensions: we measure each dimensions with some proxy variables, represented by a set of questions from the questionnaire as described in Table 3. Each question was answered on a Likert scale from 1 to 4, where 1 represents a critical situation and 4 represents the optimal situation. We measured each governance domains by the average of the response for each question at national level, as reported in Table 3 for the European level.

**Table 3:** Domains and questions from the AIR survey

Domain	Questions from the AIR survey and indicators	Medium Score
1. Political commitment	1. Do you have Regional Strategies for reducing Health Inequalities? (Question A) *No (1); No, but it is planned to be develop (2);* *Yes, included in general health strategies (3); Yes, specific strategies (4)* 2. Do you have National Strategies to reduce social health inequalities? (Question B) *No (1); No, but it is planned to be develop (2);* *Yes, included in general health strategies (3); Yes, specific strategies (4)*	2.9/4
2. Intelligence	3. Do you have evaluation instruments for measuring regional Health Inequalities? (Question C) *No (1); No, but it is planned to develop (2); Yes, integrated with other systems (3); Yes, specific system (4)* 4. Is there a mechanism for regular dissemination of the data and tools? (Question D) *No (1); No, but it is planned to disseminate (2); Yes, but not regular (3); Yes, regular dissemination (4)*	3/4
3. Accountability structures and systems	5. Does your Region have a dedicated role with key responsibilities to coordinate strategies addressing social health inequalities? (Question E) *No (1); No, but it is planned to create a role (2);* *Yes, with different department collaborating on the issue (3); Yes (4)* 6. Do you have a system to evaluate the impact of your regional strategies to reduce social health inequalities? (Question F) *No (1); No, but it is planned to be develop (2); Yes, in part (3); Yes, specific system (4)*	2.4/4
4. Policy coherence across government sectors and levels	7. Are your regional strategies linked with National Strategy on Health inequalities? (Question G) *No (1); Only in part (2); Yes, mostly (3); Yes, totally linked (4)*	2.2/4
5. Involving local people	8. Are the regional strategies implemented with other sectors/partners, along with the health sector? (Question H) *No (1); Only in part (2); Yes, mostly (3); Yes, totally (4) (considering a medium of different sectors)*	2.2/4
6. Institutional and human resource capacity	9. Who is carrying out the evaluation and measuring the impact? (Question I) *No (1); Only in part (2); Yes, mostly (3); Yes, totally (4) (considering a medium of different actors, focusing on collaboration internal and external to the health system and transparency)*	1.8/4
7. Modernized public health		
8. Learning and innovation systems	10. How have your regional institutions defined the Health Inequality Targets in their strategies? (Question J) *No (1); No, but it is planned to be develop (2); Yes, in part (3); Yes, specific system (4)* 11. Are some Health Inequality Targets relevant to the primary care settings? (Question K) *No (1); No, but it is planned to be develop (2); Yes, in part (3); Yes, specific system (4)*	2.5/4

For the purpose of the project, we use a combinations of purposive techniques involves using two of the main sampling strategies for selecting regions or cases for this research study. Firstly, the targets were identified by members of the AIR consortium [16, 17], following the opportunistic (or emerging) sample techniques for the survey [27]. Subsequently we increase the number of regions included in the sample, when a country was not represented in the consortium, using a snowballing approach by a member of a neighbor country or that share the same language. The questionnaire was sent to the health departments of the regions involved, identifying regional public administrators, regional public policy makers or other regional representatives, who have responsibility in health planning and health equity issue, with a vision of the regional and local interventions and strategies for reducing health inequalities. Each regional public administrator or policy maker was responsible to fill the questionnaire for his region. All partners involved in the project are listed in the Additional file 1.

The main difficulty in developing the questionnaire related to forming a set of questions that would take into account the different health care systems across the European countries and regions. In various AIR meetings, long discussions were held with all the partners to establish a consensus on each single question. For the countries without a data collecting system for the regional health systems, this has been done instead at the national level. To help fill the questionnaire, a glossary with the main definitions of the terms used was provided in different European languages on the basis of the literature reviewed.

A first pilot test was developed within the region leaders of the AIR Project to verify the reliability of the survey and the consistency of questions. This part of the questionnaire documented: the responsibility of the regions and countries for reducing the gap and the links with national policy and sectors; tools and data at the regional/national level to monitor health inequalities; resources dedicated to the issue; possible evaluation system and target or impact assessment.

The questionnaire was made available online from November 2010 to April 2011. Forty-seven questionnaires were received from 21 European countries (Spain, Poland, Portugal, Denmark, Austria, France, The Netherlands, United Kingdom, Hungary, Italy, Sweden, Malta, Romania, Norway, Belgium, Czech Republic, Slovene, Finland, Latvia, and Croatia) and from 47 different regions.

Of the 27 EU member states as classified in NUTS1, responses were collected from 19 member states, signifying a response rate of about 70% amongst the EU member states. Of the 271 regions in the EU as classified in NUTS2, information was collected from 47 regions, covering 17.3% of all Europeans regions.

The quality of the responses posed the main problem in the survey. Though information was collected for 47 regions, only 28 questionnaires were suitable for analysis. The reason for the heterogeneous quality and level of completeness of the surveys was that all questions were not made compulsory, to allow more freedom to the regions in responding to the individual questions. Questionnaires with a low response rate to individual questions were not considered for the analysis. In this sense, the following analysis made use of 28 questionnaires (that represents the 67% of the total sample) and they covers 13 countries. The regions considered are listed in the Table 2.

## Results

The results of the survey demonstrate that even if health inequalities become a priority for the government, and despite relevant strategies, clear targets and a system of impact assessment to demonstrate the quality and results of the actions and interventions are often missing. Considering the social determinants of health, the regional strategies implemented take into account determinants related to the demographic characteristics of the individuals such as elderly, to fragile groups (i.e. disabled people), migration, ethnic minority, and rural groups (20 of 28 regions) more than socioeconomic conditions such as living and working conditions or education (8 of 28 regions).

### The political commitment

The political commitment was generally high in all regions and at national level: the average score for all the regions considered was 2.9 out of 4. 9 of the 28 regions had specific strategies on health inequalities; 13 declared that national health strategies included strategies on health inequalities; three regions reported not having national strategies; and three had no knowledge on national strategies on health inequalities. At the regional level, the majority of regions (26 of 28) declared having strategies to reduce health inequalities, while the remaining two responded that though they did not have a strategy yet there were plans to develop the regional strategy.

### Intelligence and Accountability structures and system

The measurement and evaluation system of health inequalities was a weak point for the majority of regions owing to difficulties in collecting data and combining data on health care with data on socioeconomic conditions. In terms of intelligence, regions generally declared they have some sort of mechanism for measuring and disseminate health inequalities (score 3 out of 4). However, impact assessment in the field of health inequalities was very low: the general score of the domain dedicated to accountability was 2.4 out of 4. Only 3 of 28 regions reported they had an evaluation system. There was also a low instance of targets (such as financial incentive direct or indirect) to achieve a reduction in health inequalities. Only 9 of the 28 regions had a specific system to measure health inequalities, and 12 regions a regional system to measure health inequalities integrated with the general health measurement system. Seven declared not having a measuring system for health inequalities.

The evaluation of strategies remained a difficult issue for the health sector even though it is a necessary part of the service planning process to address health inequalities. Twelve of the 28 regions did not have any sort of impact evaluation for regional health equity strategies; 13 regions had an impact evaluation system dedicated to health strategies that includes health inequalities measures; and only three regions conducted specific impact assessment for health inequalities strategies.

### Policy coherence and involving local people

With regards the coherence across government and sectors, regions usually reported a weak connection between national and regional health equity strategies (scores 2.2 out of 4) and with other local health and social strategies (scores 2.2 out of 4). Priority settings for action and activities for the regional strategies were (a) primary care and social and community services for about 50% of the responses (14 of 28 regions), (b) hospitals, specialized services or emergency departments for about 32% (9 of 28 regions), and (c) health systems in general for the rest (5 of 28 regions).

Health promotion and prevention were the main levers to activate the reduction of health inequalities (11 of 28 regions), followed by organization of care (6 of 28 regions), funding (5 of 28 regions), and access to care (5 of 28 regions). Communication was not considered as a potential action. Regional strategies were developed with a multidisciplinary approach in 18 cases, with collaboration primarily with social services, voluntary and community sector, and ethnic minority and education sectors.

### Resource capacity and learning and innovation systems

Regions stated that the staff was not completely dedicated and trained on health equity issue (score 1.8 out of 4). The personnel responsible for carrying out the impact evaluation were health managers (11 cases), health professionals (8 cases), policy makers (6 cases), researchers (6 cases), and family doctors (2 cases). In terms of commitment to continuous improvement, only few regions declared a formal statement on performance review and targets (score 2.5 out of 4).

### Results at the national level

In this section, the survey results are discussed at the national level, considering the scores of each country of each domains and questions. Results are described in Table 3. It should be noted that the national analysis takes into account only those regions that responded to the questionnaire and thus only partially reflects the national behavior regarding health inequalities.

At the national level, only the United Kingdom had a specific national health inequalities strategy, while Austria did not have one. In the rest of the countries, the health inequalities issue was included in general health strategies. Some countries, like Spain, Austria, and Italy, indicated isolated initiatives at the regional or local levels. In most countries, the respondents reported having a regional system of measurement for monitoring health inequalities integrated with other systems. Specific measurement systems existed in Austria, Belgium, Germany, Italy, Poland, Portugal, and the United Kingdom. No regional system of evaluation was used in Slovenia, although there were plans to develop one.

Only three countries, Germany, Poland, and the United Kingdom, reported having a system to assess the impact of regional strategies for reducing inequalities in health. In most other cases, the respondents said they did not have a structured and systematic system.

The largest part of the sample indicated that the regional institutions had taken actions with the objective of assessing inequalities in terms of process, or had done so partially. Only the United Kingdom had coordinated or comprehensive strategies. France has good health inequalities strategies in regions, but it still needs an evaluation system. Not all regions in Germany and Portugal, even if they have strategies as well as regional and national targets, have a system to monitor and evaluate health inequalities and interventions. Italy, Spain, and Austria have a variable situation between different regions.

**Fig. 1:**
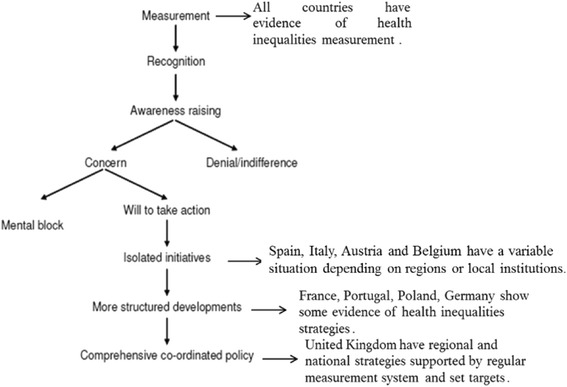
Action spectrum of inequalities and AIR survey results

Considering these results, we can also position each country in the action spectrum of health inequalities discussed by Margaret Whitehead [18, 19] on the base of the total national scores. Figure 1 describes the situation of each country in the ASI. Of the 10 countries considered, none is in a state of denial or has a mental block on tackling health inequalities. All countries have measurement systems. The 10 countries and regions are taking measures to address health inequalities with different levels of integration and evaluation of the strategies and according with results there aren’t countries that stuck in the “denial/indifference” phase, during which even if regular monitoring and airing of the issue produce little reaction Spain, Italy, and Belgium have a variable situation depending on regions; the reason could be interpreted as the absence of an integrated or clear national strategy in each country and they can be positioned at the level of “isolated initiatives”. Considering the results of the questionnaire, progress in health equity strategies at the national and regional levels seems to have been made by France, Portugal, Poland, and Germany, in terms of improving the strategies on health inequalities, and implementing both regional and national strategies. These countries show some evidence of health inequalities strategies and they can be considered in the phase of structured developments of policy on health inequalities. Finally, the United Kingdom seemed to maintain a very good policy on health equity, supported not only by regional and national strategies but also by a regular measurement system and set targets and they maintain their position in “comprehensive coordinated policy” phase.

## Discussion

The paper provides a qualitative understanding of the topics that are quantified in the Internet-based survey.

The countries profiles may indicate an increasing awareness of health inequalities at the national and regional levels, and an increasing will to take action at the regional level. Regions and countries have different approaches to addressing health equity. In line with an increasing awareness of health inequalities, only some national, regional and local health policies include objectives to decrease health inequalities. As Brown et al. underlined [14], the check list does not seek to prescribe an ideal or “best” governance structure which countries should adopt, but it can be used as a first step to analysis the level of involvement of government and policy makers.

Most regions indicate that health promotion and interventions targeted at socioeconomic disadvantaged groups are priorities. The results described in Tables 3 and 4 reflect a governmental concern for action. However, there is limited governance system and integration of strategies between national and regional levels. Moreover, priorities remain often at the level of good intentions and are not always clearly translated into specific projects. Policies and interventions are seldom evaluated.

**Table 4:** Health equity governance at national level

Countries	Question A	Question B	Question C	Question D	Question E	Question F	Question G	Question H	Question I	Question J	Question K	Total score
Austria	3.0	1.5	2.5	2.5	1.0	2.0	1.0	1.4	1.0	1.5	2.0	19
Belgium	2.7	1.0	2.7	2.3	3.3	1.3	1.3	1.5	1.3	2.7	3.3	23
The Netherlands	2.0	2.5	3.0	3.0	2.0	2.5	1.5	1.8	2.0	1.0	2.5	24
Italy	3.5	2.8	3.8	3.3	2.0	1.8	1.8	2.4	1.5	1.8	2.3	27
Poland	2.5	3.3	2.8	3.8	2.5	2.3	2.3	1.8	1.8	1.5	2.0	26
Portugal	3.5	3.3	2.5	2.3	3.3	2.3	2.8	2.5	1.8	1.8	3.0	29
Germany	3.0	3.0	2.5	2.5	2.5	2.5	2.5	2.1	2.0	2.0	4.0	29
France	3.3	3.3	2.7	2.7	3.3	2.7	3.7	2.9	2.7	3.0	3.7	34
United Kingdom	3.5	4.0	4.0	4.0	3.5	3.5	3.0	3.5	2.5	2.5	4.0	38
Spain	3	3	2.5	1.5	2	2.5	2.5	2	2	2.5	3.5	27

Good cooperation between different sectors (such as education and social sectors) and a key role for primary care, especially in health promotion, to address health inequalities are the other positive results of the survey. The lack of evidence on the scale of the effect of public health interventions, and specifically the differential effect across population groups, is a fundamental barrier to knowing what works to reduce health inequalities [28]. As Pons-Vigués M stated [29], “*Health inequalities can be tackled with appropriate health and social policies cutting across sectors, involving all community groups and governments, from local to global […]. Effective multilevel governance is clearly cross-sectoral and participative. Despite the diversity of countries’ regulations, local governments have the power to face health inequalities. Municipal sectors such as urban planning, culture, leisure, education, environment, health services, social services, housing, etc. have a clear impact on the health of the citizens*.”

There is limited evidence on the process of implementation of interventions. This is critical to the translating of evidence into practical guidance and standards that can inform wider applications [30]. Given the lack of available evidence on effective measures, the implementation of programs has encouraged local innovation and evaluation to share learning and generate new evidence [31]. Moreover, our results are similar with the European Commission [8], which suggests the need for more policy coherence in relation to the goals of Europe 2020. However, since the EU report do not consider accountability and evaluation system, we may conclude that the variability among countries and regions is wider in terms of governance to tackle health inequalities.

Table 5 summarizes positive and negative implications of information collected by the questionnaires.

**Table 5:** Positive and negative implications of the survey

Positive implications	Negative implications
Increasing awareness of health inequalities at national and regional levels	Some countries still present isolated initiatives
Increasing will to take action at national and regional levels by the policy makers	Weak evaluation system of impact of actions and interventions
Better measurement system and evidence at regional and national levels	Weakness of target quantitative approach
Good cooperation between different sectors at regional level	Poor coordination between regional and national policies

The survey highlights three mains points:

- There is still a variability of governance systems for health equity strategies both between countries and within countries; this variability encompass resources, national and local commitments and tools to tackle health inequalities.
- The proliferation of different public intervention that refers to different pathways and different target priorities; this situation may reflects the difficulties in identifying interventions that effectively reduce inequalities in health [9, 29].
- A weakness of evaluation actions and the impact of interventions in reducing inequalities, and the difficulties in having a clear and integrated vision between the national and regional levels of different strategies and results.

In this sense, our results reveal the “implementation gap” [8] between good intentions, policies and actions in terms of tackling health inequities in European Countries: the low rate of accountability and evaluation system related to strategies is a possible bias of the countries efforts since it reflect not only poor intelligence system, but also low efforts in term of resources.

### Limitations

The study had certain limitations, which should be considered. First, the regions were selected on the basis that they were involved firstly in AIR project and identified health inequalities as a theme in their public health policy documents. This implies that the results are not representative for all regions in countries. However, a snowballing system was used to select other regions in order to improve the comparison analysis. Considering this sample, the results must be discussed with these limitations in terms of representativeness. Furthermore, the aim was to identify contests and opportunity, a part from the literature review, for deeply analysing the governance system for health inequalities. In this sense, the involved regions represents a good example in discussing this issue since they can represent implicit case studies at international level. Moreover, in the best of our knowledge, there are no update studies on governance system for equity that compare different regions and countries using the Brown framework [14].

Second, the study is based mainly on the experiences of regional public policy makers and departmental managers. This implies an assumption in terms of knowledge and expertise in the health equity issue. Participants were selected firstly through an opportunistic sampling, they might not be the most representative informants in their fields or they may be more sensitive to the issue. This approach is frequently used in qualitative and descriptive analysis on equity in health that consider policy perspective [32]. However, in our study the consequently snowballing approach minimizes the risk of selection bias and improve the representativeness of the groups. Moreover, the experience of the individuals surveyed represents a good perspective in order to have a picture of the governance system across Europe, with a qualitative approach; in this sense, the results of the paper must be interpreted considering this approach and without the claim to evaluate the governance system in terms of their impacts.

As Storm et al. 2016 underline [33], the experiences of managers are crucial for the establishment and implementation of integrated health policy. In this sense, also the perspective of people involved at strategic level may reflect the complexity of the governance system at macro, meso and micro institutional level.

For these reasons, the results must be interpreted with caution and not be used to generalize the concepts. However, there are lessons learnt that may help in understanding and updating the issue of health equity strategies and in highlighting challenges and needs in order to advance for health equity.

## Conclusion

The Air project’ results highlight challenges and needs in order to advance for health equity. Progress in health equity strategies at the national and regional levels has been made by countries such as France, Portugal, Poland, and Germany. On the other hand, Spain, Italy, and Belgium have a variable situation depending on the region.

However, the results of the survey demonstrate that even if health inequalities become a priority for the government, and despite relevant strategies, clear targets and a system of impact assessment to demonstrate the quality and results of the actions and interventions are often missing. Policies to tackle inequalities in health and health care have become a marked feature of many health systems in post-industrial countries. The context and content of such policies vary markedly across these systems, reflecting different political ideologies and historical, social, and political legacies in each country, as this study demonstrated. However, recognizing the problem is not sufficient and a good governance system is needed [14] in order to strengthen the national and regional capacity system and combinations of instruments, which are capable of holding all stakeholders to account for equity results.

## Additional file

Additional file 1: List of partners, regions and countires involved in the AIR Project. (DOCX 14 kb)

## Abbreviations

Air: Adressing inequalities in health
