# Supplementary material for: Strategies and governance to reduce health inequalities: evidences from a cross-European survey
Source: Glob Health Res Policy. 2017 Jul 3;2:18. doi: 10.1186/s41256-017-0038-7 (PMC5683456; doi:10.1186/s41256-017-0038-7)
Supplement: Additional file 1: — List of partners, regions and countires involved in the AIR Project. (DOCX 14 kb) [file 41256_2017_38_MOESM1_ESM.docx]

**APPENDIX 1**

The AIR Research Group is constituted of the following.

**General coordination**

Solange Ménival (Conseil Régional d'Aquitaine, Bordeaux, France: grant holder and general coordinator), Ewelina Piznal (Conseil Régional d'Aquitaine, Bordeaux, France: project manager), Louis-Rachid Salmi (Université Bordeaux Segalen, Institut de Santé Publique, d'Epidémiologie et de Développement, Bordeaux, France: scientific coordinator).

**Literature review group**

Henri Leleu (Institut de Recherche et de Documentation en Economie de la Santé, Paris, France: coordinator), Florence Jusot, Yann Bourgueil, Marie-Odile Saton (Institut de Recherche et de Documentation en Economie de la Santé, Paris, France), Ewelina Piznal (Conseil Régional d'Aquitaine, Bordeaux, France), Ziggy Kovacs (Dekut Debreceni Kutatasfejlesztesi, Debrecen, Hungary), Andrea Novakovic (Dubrovnik Neretva County Regional Development Agency, Dubrovnik, Croatia), Antonio Daponte Codina, Julia Bolivar, Inmaculada Mateo, Isabela Ruiz Pérez (Escuela Andaluza de Salud Pública, Grenada, Spain), Vasileios Georgiou (GEORAMA, Patras, Greece), Véronique Janzyk (Hainaut Santé, Mons, Belgium), Nick Salfield (Department of Public Health in East Midlands, Notthingam, England), Zahara Ismail, Margherita Giannoni, Eleonora D'Urzo, Laura Ferialla (Poverty, Equity and Health Research Group, Terni, Italy), Sara Barsanti (Scuola Superiore Sant'Anna di Pisa, Pisa, Italy), Stephane Heijmans (ResearchLink, Thuin, Belgium), Tomasso Pucci (Tuscany Region, Florence, Italy), Paulo Di Loreto (Umbria Region, Perugia, Italy), Paris Cleanthous (University of Cyprus, Nicosia, Cyprus), Louis-Rachid Salmi, Evelyne Mouillet (Université Bordeaux Segalen, Institut de Santé Publique, d'Epidémiologie et de Développement, Bordeaux, France).

**Survey group**

Sara Barsanti (Scuola Superiore Sant'Anna di Pisa, Laboratorio Management e Sanità, Pisa, Italy: coordinator), Nicola Iacovina and Sabina Nuti (Scuola Superiore Sant'Anna di Pisa, Laboratorio Management e Sanità, Pisa, Italy), Emilie de Saint Pol (Agence Régionale de Santé d'Aquitaine, Bordeaux, France), Ewelina Piznal (Conseil Régional d'Aquitaine, Bordeaux, France), Nick Salfield (Department of Public Health in East Midlands, Notthingam, England), Zsuzana Szabo and Ziggy Kovacs (Dekut Debreceni Kutatasfejlesztesi, Debrecen, Hungary), Andrea Novakovic, Matija Cale Mratovic and Ankica Dzona Boban (Dunea and Public Health Institute in Dubrovnic-Neretva County, Dubrovnic, Croatia), Antonio Daponte, Julia Bolivar, Inmaculada Mateo and Isabela Ruiz Pérez (Escuela Andaluza de Salud Pública, Granada, Spain), Floris Barnhoorn (European Public Health Association, Utrecht, Netherlands), Luc Berghmans and Julie Harlet (Hainaut Santé, Mons, Belgium), Yann Bourgueil (Institut de Recherche et de Documentation en Economie de la Santé, Paris, France), Krzysztof Bederski (John Paul II Hospital in Krakow, Krakow, Poland), Gabriele Theren (Ministry of Health and Work Affairs Saxony-Anhalt, Magdeburg, Germany), Neville Caleja (Ministry for Social Policy, Health, Elderly, Community Care, G'Mangia, Malta), Mariana Almeida (Regional Health Administration in Algarve, Faro, Portugal), Stephane Heijmans (ResearchLink, Thuin, Belgium), Soledad Marquez and Ana Carriazo (Consejeria de Salud, Junta de Andalucia, Sevilla, Spain), Ingrid Gonzalez-Seco (Servicio Andaluz de Salud, Sevilla, Spain), Louis-Rachid Salmi (Université Bordeaux Segalen, Institut de Santé Publique, d'Epidémiologie et de Développement, Bordeaux, France), Arnd Hofmeister (University of Applied Sciences Magdebourg-Stendal, Magdeburg, Germany), Paris Cleanthous (University of Cyprus, Nicosia, Cyprus).

**Assessment of interventions**

Antonio Daponte (Escuela Andaluza de Salud Pública, Grenada, Spain: coordinator), Julia Bolivar, Inmaculada Mateo, Isabel Ruiz Pérez and Mariola Bernal Solano (Escuela Andaluza de Salud Pública, Grenada, Spain), Soledad Marquez (Consejeria de Salud, Junta de Andalucia, Sevilla, Spain), Ingrid González-Seco (Servicio Andaluz de Salud, Sevilla, Spain), Emilie de Saint Pol (Agence Régionale de Santé d'Aquitaine, Bordeaux, France), Ewelina Piznal (Conseil Régional d'Aquitaine, Bordeaux, France), Ann Godwin (Department of Public Health in East Midlands, Nottingham, England), Matija Cale Mratovic (Dunea and Public Health Institute in Dubrovnic-Neretva County, Dubrovnic, Croatia), Efstratia Mourtou (GEORAMA, Patras, Greece), Luc Berghmans (Hainaut Santé, Mons, Belgium), Yann Bourgueil (Institut de Recherche et de Documentation en Economie de la Santé, Paris, France), Gabriele Theren and Thomas Hering (Ministry of Health and Work Affairs Saxony-Anhalt, Magdeburg, Germany), Stephane Heijmans (ResearchLink, Thuin, Belgium), Sara Barsanti (Scuola Superiore Sant'Anna di Pisa, Laboratorio Management e Sanità, Pisa, Italy), Louis-Rachid Salmi (Université Bordeaux Segalen, Institut de Santé Publique, d'Epidémiologie et de Développement, Bordeaux, France).

**Associated partners of the air project**

Conseil Régional d'Aquitaine (Bordeaux, France): Ménival S (general coordination), Piznal E (coordination, survey, assessment of interventions, dissemination); Université Bordeaux Segalen, Institut de Santé Publique, d'Epidémiologie et de Développement (Bordeaux, France): Salmi LR (general coordination, survey, assessment of interventions, evaluation of project, recommendations, dissemination), Mouillet E (literature review, dissemination); Hainaut Santé (Mons, Belgium): Berghmans L (survey, evaluation of interventions, recommendations, dissemination), Harlet J (survey, dissemination), Bizel P (dissemination), Pensis G (dissemination); Dekut Debreceni Kutatasfejlesztesi (Debrecen, Hungary): Szabo Z (evaluation of the project, dissemination), Kovacs Z (evaluation of the project, dissemination); Institut de Recherche et de Documentation en Economie de la Santé (Paris, France): Bourgueil Y (literature review, survey, evaluation of the interventions, recommendations, dissemination), Jusot F (literature review), Leleu H (literature review); Scuola Superiore Sant'Anna di Pisa, Laboratorio Management e Sanità (Pisa, Italy): Barsanti S (survey, evaluation of the interventions, recommendations, disseminations), Iacovina N (survey); Escuela Andaluza de Salud Pública (Granada, Spain): Daponte A (survey, selection and evaluation of the interventions, recommendations, dissemination), Bolivar J (survey, selection and evaluation of the interventions, recommendations, dissemination), Bernal Solano M (survey, selection and evaluation of interventions, recommendations, dissemination), Mateo I (survey, selection and evaluation of the interventions, recommendations, dissemination), Ruis Pérez I (survey, selection and evaluation of interventions, recommendations, dissemination); Department of Public Health in East Midlands, East Midlands Strategic Health Authority (Notthingam, England): Salfield N (survey, dissemination), Godwin A (evaluation of interventions), Rajaratnam G (recommendations), Jobarteh J (dissemination); University of Cyprus (Nicosia, Cyprus): Cleanthous P (survey, dissemination); ResearchLink (Thuin Belgium): Heijmans S (literature review, survey, evaluation of interventions, dissemination); Dunea and Public Health Institute in Dubrovnic-Neretva County (Dubrovnik, Croatia): Andrea Novakovic (dissemination), Matija Cale Mratovic (survey, evaluation of interventions, dissemination), Ankica Dzona-Boban (survey, dissemination); Poverty, Equity, and Health Research Group (Terni, Italy): Ismail Z (literature review), Giannoni M (literature review, survey, dissemination), D'Urzo E (literature review, survey, dissemination), Ferialla L (literature review, survey, dissemination); Umbria Region (Perugia, Italy): Cassucci P (dissemination); Tuscany Region (Florence, Italy): Ammannati B, Tanini D, Bottai R and Berti A (dissemination); GEORAMA (Patras, Greece): Georgiou V (dissemination).

**Collaborative partners**

County Council (Gävleborg, Sweden): Eva Hultgren; European Public Health Association (Utrecht, Netherlands): Barnhoorn F; Agence Régionale de Santé (Bordeaux, France): de Saint Pol E (survey, evaluation of the interventions, dissemination); Lincolnshire Primary Care Trust (Lincoln, England): Mc Shane M; Servicio Andaluz de Salud (Sevilla, Spain): Gonzalez-Seco I (survey, evaluation of the interventions); Union Régionale des Professionnels de Santé d'Aquitaine (Bordeaux, France): Guérin D; Leicestershire County Council and Rutland PCT (Leicester, England), Wardle M; Debrecen University (Pecs, Hungary): Sandor J; Ministry of Health and Work Affairs Saxony-Anhalt (Magdeburg, Germany): Theren G (survey, evaluation of the intervention, dissemination); University of Applied Sciences Magdebourg-Stendal (Magdeburg, Germany): Hofmeister A (survey, dissemination); Consejeria de Salud, Junta de Andalucia (Sevilla, Spain): Carriazo A (survey, dissemination), Marquez S (survey, evaluation of interventions, dissemination); Research centre in behaviour and social issues (Nicosia, Cyprus): Christina Loizou; John Paul II Hospital in Krakow (Krakow, Poland): Bederski K (survey, dissemination): Ministry for Social Policy, Health, Elderly, Community Care (G'Mangia, Malta) Caleja N (survey, dissemination); Regional Health Administration in Algarve (Faro, Portugal): Almeida M (survey, dissemination).
